# Methyl 2-[(*Z*)-5-methyl-2-oxoindolin-3-yl­idene]hydrazinecarbodi­thio­ate

**DOI:** 10.1107/S2414314624009672

**Published:** 2024-10-08

**Authors:** Mohd Abdul Fatah Abdul Manan, David B. Cordes, Aidan P. McKay

**Affiliations:** a Faculty of Applied Sciences, Universiti Teknologi MARA, 40450 Shah Alam, Selangor, Malaysia; b EaStCHEM School of Chemistry, University of St Andrews, St Andrews, Fife KY16 9ST, United Kingdom; University of Aberdeen, United Kingdom

**Keywords:** crystal structure, di­thio­carbazate, 5-methyl­isatin, C=O⋯C=O inter­action

## Abstract

The title di­thio­carbazate imine, C_11_H_11_N_3_OS_2_, was obtained from the condensation reaction of *S*-methyl­dithio­carbazate (SMDTC) and 5-methyl­isatin. It shows a *Z* configuration about the imine C=N bond, which is associated with an intra­molecular N—H⋯O hydrogen bond that closes an *S*(6) ring. In the crystal, inversion dimers linked by pairwise N—H⋯O hydrogen bonds generate *R*^2^_2_(8) loops. The extended structure features C—H⋯S contacts as well as reciprocal carbon­yl–carbonyl (C=O⋯C=O) inter­actions.

## Abstract 2

The crystal structure of a new *S*-methyl substituted di­thio­carbazate imine containing the 5-methyl­isatin moiety is described.

## Graphical abstract

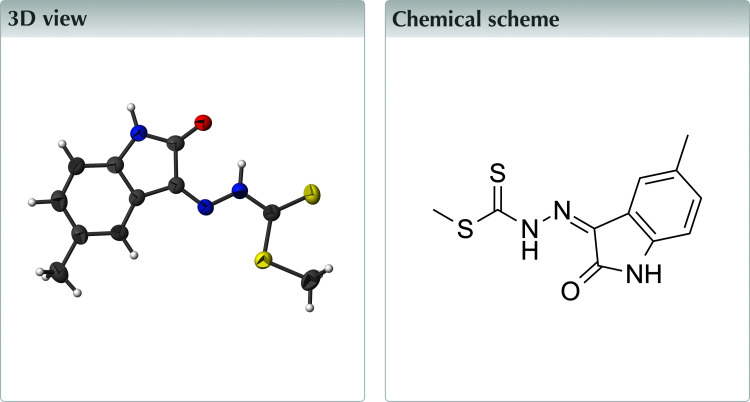

## Structure description

In medicinal chemistry, isatin (1*H*-indole-2,3-dione, C_8_H_5_NO_2_) and its derivatives represent an important class of heterocyclic compounds with potential pharmacological properties (Shu *et al.*, 2024 ▸). Taking advantage of the versatile reactivity of the isatin nucleus, a huge library of isatin derivatives with various applications is now available. Most of these derivatives have been obtained by utilizing either the high reactivity of its 3-carbonyl group or the nucleophilic nature of its NH group. The NH group can undergo *N*-acyl­ation, *N*-aryl­ation or *N*-alkyl­ation, whereas the C3 carbonyl group can be utilized in the synthesis of hydrazone or imine derivatives as well as oxindoles and spiro­cyclic compounds (Nath *et al.*, 2020 ▸). These derivatives are reported to possess several bio­logical activities and find applications in the field of crystal engineering, supra­molecular chemistry and materials science (Mehreen *et al.*, 2022*a* ▸; Ahmed *et al.*, 2019 ▸).

Recently, chemists have recognized both one-sided and reciprocal carbon­yl–carbonyl inter­actions as non-covalent inter­actions of significant inter­est due to their ability to influence the geometries of small mol­ecules and affect the three dimensional structures of peptides, peptoids, proteins and polyesters (Rahim *et al.*, 2017 ▸). Very recently, the use of isatin-derived compounds as potent α-glucosidase inhibitors in managing diabetes has been reported, highlighting the role of C=O⋯C=O inter­actions in inhibiting α-glucosidase and controlling postprandial hyperglycemia (Mehreen *et al.*, 2022*b* ▸). As a continuation of our research inter­ests in isatin derivatives, we now report the synthesis and crystal structure of the title compound, C_11_H_11_N_3_OS_2_.

**Figure 1:**
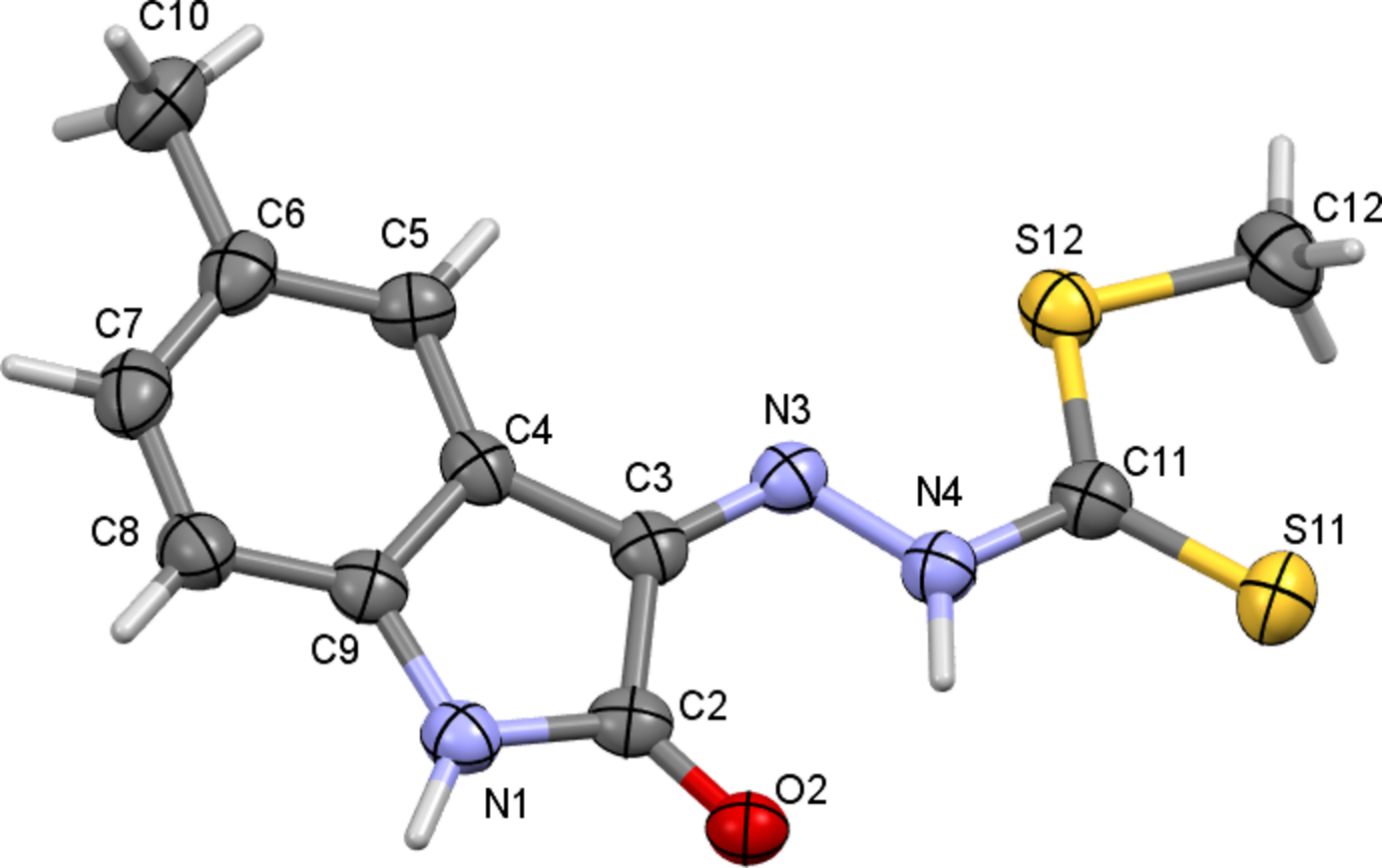
The mol­ecular structure of the title compound, showing displacement ellipsoids drawn at the 50% probability level.

The asymmetric unit of the title compound (Fig. 1 ▸) comprises one mol­ecule and crystallizes in the monoclinic space group *P*2_1_/*c*. The methyl hydrazinecarbodi­thio­ate chain connects to the nine-membered 5-methyisatin ring at C3 and adopts a near planar geometry (r.m.s. deviation from planarity = 0.033 Å). The essentially planar conformation of the mol­ecule is associated with the formation of an intra­molecular N4—H4⋯O2 hydrogen bond (Table 1 ▸), which closes an *S*(6) loop. In the solid state, the compound exists in its thione tautomeric form with the di­thio­carbazate fragment adopting a *Z* conformation about the C=N bond with respect to the 5-methyl­isatin moiety, while the *S*-methyl group adopts a *syn* conformation relative to the azomethine nitro­gen atom. Otherwise, the bond lengths and angles in the title compound may be regarded as normal.

**Figure 2:**
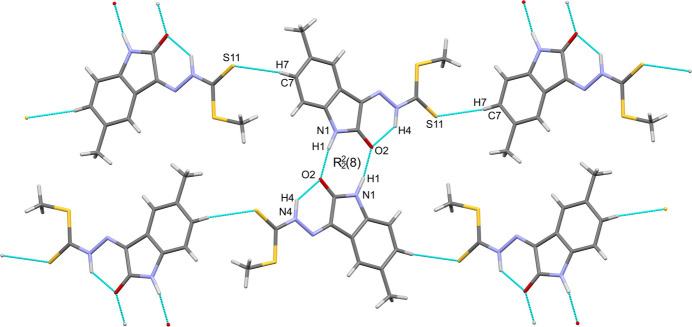
View of the N—H⋯O and C—H⋯S hydrogen bonds generating 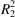
(8) dimers (centre) and C(10) chains (left to right), which combine to form corrugated (102) sheets.

**Figure 3:**
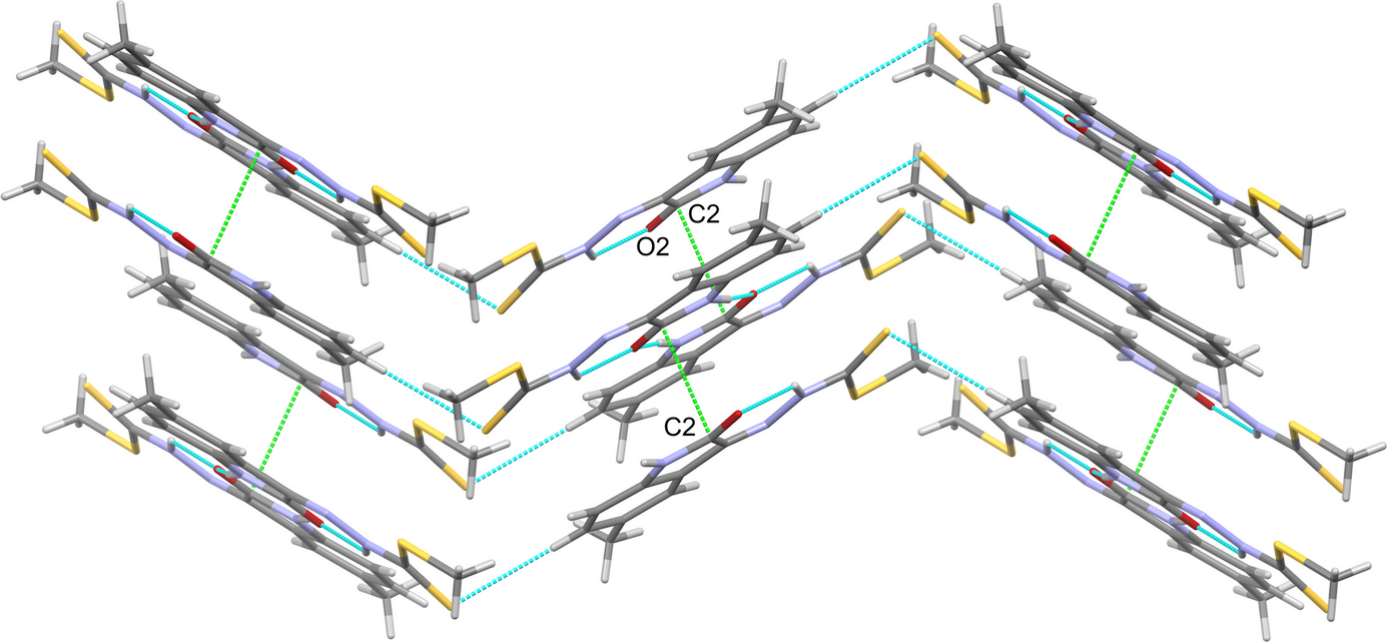
View showing the stacking of the corrugated sheets supported by reciprocal carbon­yl–carbonyl inter­actions (green).

**Figure 4:**
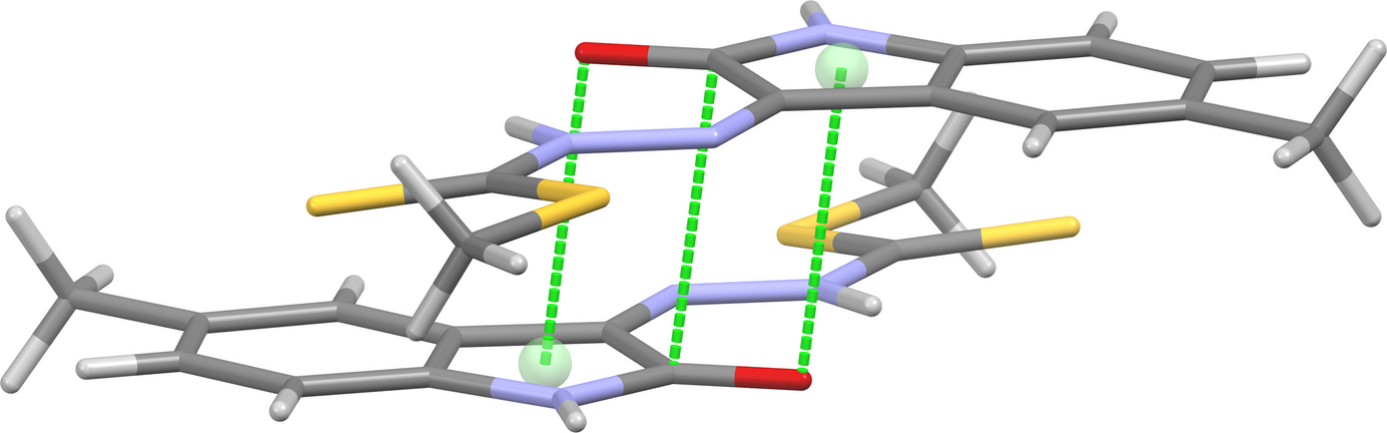
Offset geometry of the carbon­yl–carbonyl inter­action showing how O2 is positioned over the adjacent pyrrolone ring.

In the crystal, the mol­ecules of the title compound form inversion dimers through pairwise N1—H1⋯O2 hydrogen bonds (Table 1 ▸) in the common 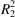
(8) motif. There are additional weak, non-classical C7—H7⋯S11 hydrogen bonds, which link mol­ecules into *C*(10) chains propagating along [010]. The combination of the chains and inversion dimers forms corrugated sheets lying in the (102) plane (Fig. 2 ▸). The aforementioned sheets stack by way of reciprocal carbon­yl–carbonyl inter­actions [C2⋯O2 = 3.166 (6) Å, C=O⋯C = 75.1 (3)°, O=C⋯O = 104.8 (3)°] (Fig. 3 ▸). The contact observed differs from the ideal motif-II type inter­action (Sahariah & Sarma, 2019 ▸) with O2 lying over the adjacent pyrrolone ring (Fig. 4 ▸).

## Synthesis and crystallization

The di­thio­carbazate precursor (SMDTC) was prepared by the literature method (Das & Livingstone, 1976 ▸). The title compound was prepared by adding 5-methyl­isatin (1.61 g, 10.0 mmol, 1.0 eq) dissolved in hot ethanol (20 ml) to a solution of SMDTC (1.22 g, 10.0 mmol, 1.0 eq) in hot ethanol (35 ml). The mixture was heated (80°C) with continuous stirring for 15 min and later allowed to stand for 20 min at room temperature until a precipitate formed, which was then filtered and dried over silica gel, yielding orange needles of the title compound on recrystallization form ethanol solution (yield: 2.12 g, 80%). m.p. 236–237°C; ^1^H NMR (400 MHz, *d*_6_-DMSO) δ: (p.p.m): 2.31 (*s*, 3H), 2.62 (*s*, 3H), 6.84 (*d*, *J* = 7.96 Hz, 1H), 7.22 (*d*, *J* = 7.96 Hz, 1H), 7.36 (*s*, 1H), 11.27 (*s*, 1H), 14.00 (*s*, 1H); HRMS *m*/*z* (ESI^+^), found: [*M*+H]^+^ 266.0417, C_11_H_12_N_3_OS_2_ requires [*M*+H]^+^ 266.0422.

## Refinement

Crystal data, data collection and structure refinement details are summarized in Table 2 ▸. The structure was refined as a two-component twin with component 2 rotated by 2.05° around [001] (reciprocal) or [105] (direct), and a refined twin fraction of 0.128 (6).

## Supplementary Material

Crystal structure: contains datablock(s) I. DOI: 10.1107/S2414314624009672/hb4486sup1.cif

Structure factors: contains datablock(s) I. DOI: 10.1107/S2414314624009672/hb4486Isup2.hkl

Supporting information file. DOI: 10.1107/S2414314624009672/hb4486Isup3.cml

CCDC reference: 2388258

Additional supporting information:  crystallographic information; 3D view; checkCIF report

## Figures and Tables

**Table 1:** Hydrogen-bond geometry (Å, °)

*D*—H⋯*A*	*D*—H	H⋯*A*	*D*⋯*A*	*D*—H⋯*A*
N4—H4⋯O2	0.98 (2)	2.01 (6)	2.754 (6)	130 (6)
N1—H1⋯O2^i^	0.98 (2)	1.85 (2)	2.825 (6)	171 (7)

Symmetry code: (i) 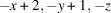.

**Table 2:** Experimental details

Crystal data	
Chemical formula	C_11_H_11_N_3_OS_2_
*M*_r_	265.35
Crystal system, space group	Monoclinic, *P*2_1_/*c*
Temperature (K)	125
*a*, *b*, *c* (Å)	4.9897 (4), 21.8014 (19), 11.3394 (9)
β (°)	92.995 (8)
*V* (Å^3^)	1231.83 (18)
*Z*	4
Radiation type	Cu *K*α
μ (mm^−1^)	3.82
Crystal size (mm)	0.23 × 0.01 × 0.01

Data collection	
Diffractometer	Rigaku XtaLAB P200K
Absorption correction	Multi-scan (*CrysAlis PRO*; Rigaku OD, 2023 ▸)
*T*_min_, *T*_max_	0.631, 1.000
No. of measured, independent and observed [*I* > 2σ(*I*)] reflections	23143, 2554, 1548
*R*_int_	0.171
(sin θ/λ)_max_ (Å^−1^)	0.630

Refinement	
*R*[*F*^2^ > 2σ(*F*^2^)], *wR*(*F*^2^), *S*	0.079, 0.185, 1.09
No. of reflections	2554
No. of parameters	165
No. of restraints	2
H-atom treatment	H atoms treated by a mixture of independent and constrained refinement
Δρ_max_, Δρ_min_ (e Å^−3^)	0.46, −0.63

Computer programs: *CrysAlis PRO* (Rigaku OD, 2023 ▸), *SHELXT* (Sheldrick, 2015*a* ▸), *SHELXL2019/3* (Sheldrick, 2015*b* ▸), *Mercury* (Macrae *et al.*, 2020 ▸), *OLEX2* (Dolomanov *et al.*, 2009 ▸) and *publCIF* (Westrip, 2010 ▸).

## full crystallographic data

### Methyl 2-[(Z)-5-methyl-2-oxoindolin-3-ylidene]hydrazinecarbodithioate Crystal data

**Table:**

C_11_H_11_N_3_OS_2_	*F*(000) = 552
*M**_r_* = 265.35	*D*_x_ = 1.431 Mg m^−^^3^
Monoclinic, *P*2_1_/*c*	Cu *K*α radiation, λ = 1.54184 Å
*a* = 4.9897 (4) Å	Cell parameters from 2965 reflections
*b* = 21.8014 (19) Å	θ = 4.0–68.3°
*c* = 11.3394 (9) Å	µ = 3.82 mm^−^^1^
β = 92.995 (8)°	*T* = 125 K
*V* = 1231.83 (18) Å^3^	Needle, orange
*Z* = 4	0.23 × 0.01 × 0.01 mm

### Methyl 2-[(Z)-5-methyl-2-oxoindolin-3-ylidene]hydrazinecarbodithioate Data collection

**Table:**

Rigaku XtaLAB P200K diffractometer	2554 independent reflections
Radiation source: Rotating Anode, Rigaku MM-007HF	1548 reflections with *I* > 2σ(*I*)
Rigaku Osmic Confocal Optical System monochromator	*R*_int_ = 0.171
Detector resolution: 5.8140 pixels mm^-1^	θ_max_ = 76.3°, θ_min_ = 4.1°
shutterless scans	*h* = −6→6
Absorption correction: multi-scan (CrysAlisPro; Rigaku OD, 2023)	*k* = −27→27
*T*_min_ = 0.631, *T*_max_ = 1.000	*l* = 0→14
23143 measured reflections	

### Methyl 2-[(Z)-5-methyl-2-oxoindolin-3-ylidene]hydrazinecarbodithioate Refinement

**Table:**

Refinement on *F*^2^	Primary atom site location: dual
Least-squares matrix: full	Hydrogen site location: mixed
*R*[*F*^2^ > 2σ(*F*^2^)] = 0.079	H atoms treated by a mixture of independent and constrained refinement
*wR*(*F*^2^) = 0.185	*w* = 1/[σ^2^(*F*_o_^2^) + 4.5988*P*] where *P* = (*F*_o_^2^ + 2*F*_c_^2^)/3
*S* = 1.09	(Δ/σ)_max_ < 0.001
2554 reflections	Δρ_max_ = 0.46 e Å^−^^3^
165 parameters	Δρ_min_ = −0.63 e Å^−^^3^
2 restraints	

### Methyl 2-[(Z)-5-methyl-2-oxoindolin-3-ylidene]hydrazinecarbodithioate Special details

**Table:**

Geometry. All esds (except the esd in the dihedral angle between two l.s. planes) are estimated using the full covariance matrix. The cell esds are taken into account individually in the estimation of esds in distances, angles and torsion angles; correlations between esds in cell parameters are only used when they are defined by crystal symmetry. An approximate (isotropic) treatment of cell esds is used for estimating esds involving l.s. planes.
Refinement. Refined as a 2-component twin using an HKLF5 file generated by TWINROTMAT running in PLATON (Spek, 2009), with twin law [-1 0 0 0 -1 0 0.237 0 1]. N—H hydrogen atoms located from F_map_ and refined isotropically with appropriate distance restraints.The N-bound H atoms were located in a difference map and refined isotropically with a distance restraint. The C-bound H atoms were located geometrically (phenyl C—H =0.95 Å, methyl C—H = 0.98 Å) and refined as riding atoms. The constraint *U*_iso_(H) = 1.2*U*_eq_(phenyl C) or 1.5*U*_eq_(methyl C) was applied in all cases.

### Methyl 2-[(Z)-5-methyl-2-oxoindolin-3-ylidene]hydrazinecarbodithioate Fractional atomic coordinates and isotropic or equivalent isotropic displacement parameters (Å^2^)

**Table:**

	*x*	*y*	*z*	*U*_iso_*/*U*_eq_	
S11	0.1241 (3)	0.27752 (7)	−0.01332 (14)	0.0469 (4)	
S12	−0.0419 (3)	0.32382 (7)	0.22526 (13)	0.0413 (4)	
O2	0.7445 (7)	0.43673 (16)	0.0099 (3)	0.0370 (9)	
N1	0.8229 (9)	0.5243 (2)	0.1228 (4)	0.0368 (11)	
H1	0.962 (11)	0.542 (3)	0.075 (6)	0.09 (3)*	
N3	0.3151 (9)	0.4171 (2)	0.1850 (4)	0.0333 (10)	
N4	0.3199 (9)	0.3741 (2)	0.0983 (4)	0.0348 (10)	
H4	0.440 (12)	0.378 (3)	0.033 (5)	0.09 (3)*	
C2	0.6980 (10)	0.4710 (2)	0.0933 (5)	0.0332 (12)	
C3	0.4865 (10)	0.4614 (2)	0.1812 (5)	0.0325 (12)	
C4	0.5034 (10)	0.5136 (2)	0.2617 (5)	0.0330 (12)	
C5	0.3648 (11)	0.5306 (2)	0.3595 (5)	0.0364 (12)	
H5	0.227148	0.505107	0.387426	0.044*	
C6	0.4315 (12)	0.5858 (3)	0.4162 (5)	0.0410 (14)	
C7	0.6350 (12)	0.6224 (3)	0.3727 (6)	0.0460 (15)	
H7	0.678915	0.659976	0.411573	0.055*	
C8	0.7741 (12)	0.6058 (3)	0.2752 (5)	0.0430 (14)	
H8	0.909675	0.631488	0.246180	0.052*	
C9	0.7089 (11)	0.5509 (2)	0.2222 (5)	0.0354 (12)	
C10	0.2876 (13)	0.6062 (3)	0.5235 (5)	0.0503 (16)	
H10A	0.195041	0.645094	0.506230	0.075*	
H10B	0.418146	0.611821	0.590187	0.075*	
H10C	0.156207	0.574984	0.543505	0.075*	
C11	0.1427 (11)	0.3261 (3)	0.0983 (5)	0.0363 (12)	
C12	−0.2434 (12)	0.2564 (3)	0.1973 (6)	0.0517 (16)	
H12A	−0.344567	0.260750	0.121366	0.078*	
H12B	−0.368701	0.251475	0.260409	0.078*	
H12C	−0.126802	0.220314	0.195053	0.078*	

### Methyl 2-[(Z)-5-methyl-2-oxoindolin-3-ylidene]hydrazinecarbodithioate Atomic displacement parameters (Å^2^)

**Table:**

	*U*^11^	*U*^22^	*U*^33^	*U*^12^	*U*^13^	*U*^23^
S11	0.0550 (9)	0.0417 (8)	0.0435 (8)	0.0003 (7)	−0.0028 (7)	−0.0055 (7)
S12	0.0400 (8)	0.0386 (8)	0.0453 (8)	−0.0014 (6)	0.0031 (6)	0.0030 (7)
O2	0.035 (2)	0.038 (2)	0.037 (2)	0.0033 (17)	0.0029 (17)	0.0032 (17)
N1	0.033 (2)	0.035 (2)	0.043 (3)	0.002 (2)	0.002 (2)	0.003 (2)
N3	0.034 (2)	0.033 (2)	0.033 (2)	0.004 (2)	0.0005 (19)	0.0010 (19)
N4	0.035 (2)	0.034 (2)	0.036 (3)	0.001 (2)	0.002 (2)	0.001 (2)
C2	0.031 (3)	0.035 (3)	0.034 (3)	0.008 (2)	0.000 (2)	0.009 (2)
C3	0.031 (3)	0.031 (3)	0.035 (3)	0.005 (2)	−0.004 (2)	0.005 (2)
C4	0.030 (3)	0.031 (3)	0.037 (3)	0.001 (2)	−0.004 (2)	0.006 (2)
C5	0.039 (3)	0.038 (3)	0.033 (3)	0.003 (2)	0.004 (2)	0.006 (2)
C6	0.044 (3)	0.034 (3)	0.045 (3)	0.009 (3)	−0.004 (3)	−0.003 (3)
C7	0.044 (3)	0.039 (3)	0.055 (4)	0.008 (3)	−0.009 (3)	−0.008 (3)
C8	0.037 (3)	0.037 (3)	0.055 (4)	−0.001 (3)	0.004 (3)	−0.002 (3)
C9	0.032 (3)	0.037 (3)	0.036 (3)	0.005 (2)	−0.003 (2)	0.004 (2)
C10	0.063 (4)	0.045 (3)	0.043 (3)	0.010 (3)	0.001 (3)	−0.005 (3)
C11	0.037 (3)	0.034 (3)	0.038 (3)	0.003 (2)	0.001 (2)	0.006 (2)
C12	0.045 (4)	0.036 (3)	0.074 (5)	−0.002 (3)	−0.001 (3)	0.010 (3)

### Methyl 2-[(Z)-5-methyl-2-oxoindolin-3-ylidene]hydrazinecarbodithioate Geometric parameters (Å, º)

**Table:**

S11—C11	1.649 (6)	C5—H5	0.9500
S12—C11	1.750 (6)	C5—C6	1.396 (8)
S12—C12	1.799 (6)	C6—C7	1.402 (8)
O2—C2	1.237 (6)	C6—C10	1.511 (8)
N1—H1	0.98 (2)	C7—H7	0.9500
N1—C2	1.354 (7)	C7—C8	1.384 (8)
N1—C9	1.412 (7)	C8—H8	0.9500
N3—N4	1.360 (6)	C8—C9	1.372 (8)
N3—C3	1.292 (7)	C10—H10A	0.9800
N4—H4	0.98 (2)	C10—H10B	0.9800
N4—C11	1.370 (7)	C10—H10C	0.9800
C2—C3	1.503 (7)	C12—H12A	0.9800
C3—C4	1.460 (7)	C12—H12B	0.9800
C4—C5	1.387 (7)	C12—H12C	0.9800
C4—C9	1.400 (7)		
			
C11—S12—C12	101.1 (3)	C8—C7—C6	122.3 (6)
C2—N1—H1	122 (5)	C8—C7—H7	118.8
C2—N1—C9	110.5 (4)	C7—C8—H8	121.3
C9—N1—H1	128 (5)	C9—C8—C7	117.4 (5)
C3—N3—N4	117.0 (4)	C9—C8—H8	121.3
N3—N4—H4	121 (4)	C4—C9—N1	110.5 (5)
N3—N4—C11	119.3 (4)	C8—C9—N1	127.7 (5)
C11—N4—H4	119 (4)	C8—C9—C4	121.8 (5)
O2—C2—N1	127.3 (5)	C6—C10—H10A	109.5
O2—C2—C3	126.1 (5)	C6—C10—H10B	109.5
N1—C2—C3	106.6 (5)	C6—C10—H10C	109.5
N3—C3—C2	128.0 (5)	H10A—C10—H10B	109.5
N3—C3—C4	125.4 (5)	H10A—C10—H10C	109.5
C4—C3—C2	106.6 (4)	H10B—C10—H10C	109.5
C5—C4—C3	133.9 (5)	S11—C11—S12	127.0 (3)
C5—C4—C9	120.4 (5)	N4—C11—S11	120.0 (4)
C9—C4—C3	105.8 (5)	N4—C11—S12	112.9 (4)
C4—C5—H5	120.6	S12—C12—H12A	109.5
C4—C5—C6	118.7 (5)	S12—C12—H12B	109.5
C6—C5—H5	120.6	S12—C12—H12C	109.5
C5—C6—C7	119.3 (5)	H12A—C12—H12B	109.5
C5—C6—C10	120.8 (5)	H12A—C12—H12C	109.5
C7—C6—C10	119.9 (5)	H12B—C12—H12C	109.5
C6—C7—H7	118.8		
			
O2—C2—C3—N3	−1.0 (8)	C3—C4—C9—N1	−0.8 (6)
O2—C2—C3—C4	−179.2 (5)	C3—C4—C9—C8	177.6 (5)
N1—C2—C3—N3	178.2 (5)	C4—C5—C6—C7	0.2 (8)
N1—C2—C3—C4	0.1 (5)	C4—C5—C6—C10	−179.7 (5)
N3—N4—C11—S11	173.7 (4)	C5—C4—C9—N1	179.3 (5)
N3—N4—C11—S12	−7.1 (6)	C5—C4—C9—C8	−2.3 (8)
N3—C3—C4—C5	2.1 (9)	C5—C6—C7—C8	−0.2 (9)
N3—C3—C4—C9	−177.7 (5)	C6—C7—C8—C9	−1.0 (9)
N4—N3—C3—C2	−1.5 (7)	C7—C8—C9—N1	−179.7 (5)
N4—N3—C3—C4	176.3 (5)	C7—C8—C9—C4	2.2 (8)
C2—N1—C9—C4	0.9 (6)	C9—N1—C2—O2	178.6 (5)
C2—N1—C9—C8	−177.3 (5)	C9—N1—C2—C3	−0.6 (5)
C2—C3—C4—C5	−179.7 (5)	C9—C4—C5—C6	1.0 (8)
C2—C3—C4—C9	0.5 (5)	C10—C6—C7—C8	179.7 (5)
C3—N3—N4—C11	179.9 (5)	C12—S12—C11—S11	0.1 (5)
C3—C4—C5—C6	−178.8 (5)	C12—S12—C11—N4	−179.0 (4)

### Methyl 2-[(Z)-5-methyl-2-oxoindolin-3-ylidene]hydrazinecarbodithioate Hydrogen-bond geometry (Å, º)

**Table:**

*D*—H···*A*	*D*—H	H···*A*	*D*···*A*	*D*—H···*A*
N4—H4···O2	0.98 (2)	2.01 (6)	2.754 (6)	130 (6)
N1—H1···O2^i^	0.98 (2)	1.85 (2)	2.825 (6)	171 (7)

Symmetry code: (i) −*x*+2, −*y*+1, −*z*.
